# Changing Pattern of Oral Cavity Lesions and Personal Habits Over a Decade: Hospital Based Record Analysis from Allahabad

**DOI:** 10.4103/0970-0218.58391

**Published:** 2009-10

**Authors:** Vatsala Misra, Premala A Singh, Nirupama Lal, Pooja Agarwal, Mamta Singh

**Affiliations:** Department of Pathology, Moti Lal Nehru Medical College, University of Allahabad, India

**Keywords:** Oral cavity, Oral submucous fibrosis (OSMF), squamous cell carcinoma, tobacco

## Abstract

**Aim:**

To do a prospective clinicohistological study of premalignant and malignant lesions of the oral cavity, and compare it with a 10-year retrospective data, especially in terms of incidence, age distribution, personal habits, and site and type of lesion.

**Material and Methods:**

Sections from 776 lesions of the oral cavity, which included 647 lesions of a 10-year (1993 – 2003) retrospective study and 129 lesions of a one-year (2003 – 2004) prospective study, were observed clinically, and a histological correlation was carried out.

**Results:**

Premalignant lesions included 78 cases of leukoplakia, 68 cases of oral submucous fibrosis, and 76 cases of squamous papilloma. Their incidence has increased in the last decade from 0.15 to 0.53. These lesions commonly presented in the fourth decade of life, as white patches in leukoplakia and oral submucous fibrosis, and as a growth in squamous cell papilloma. Squamous cell carcinoma was the commonest lesion (57%). Its incidence has increased significantly in the last decade. The mean age of presentation was the sixth decade. A personal history of tobacco chewing was given by most of the patients in the retrospective group, while the use of pan masala was found to be maximum in the prospective group. The overall agreement between the clinical and histological diagnosis was 95.36% (740 / 776) and the kappa coefficient of agreement was 0.9256.

**Conclusion:**

Histology along with a detailed clinical workup was found to be a useful, reliable, and accurate diagnostic technique for lesions of the oral cavity. An increase in premalignant lesions in the prospective study, associated with increased pan masala intake is alarming and needs to be taken care of.

## Introduction

Although the oral cavity lesions constitute only a small minority of pathological conditions, they are of great significance, as they have a potential to jeopardize the health and longevity of the patient.(1) Globally, about, 5,75,000 new cases and 3,20,000 deaths occur every year from oral cancer.(2) It is essential to establish an accurate diagnosis, to initiate optimal therapy for oral cavity lesions. An adequate incision biopsy taken from an area representative of the lesion can provide over 98% diagnostic accuracy as to whether the lesion is malignant or not, when routine pathological techniques are used.(3) Most oropharyngeal cancers in India present in advanced stages of malignancy.

The present study was undertaken with a view to study various lesions of the oral cavity, especially premalignant lesions, and correlate the histological changes with relevant clinical findings. An effort has also been made to compare the retrospective data with the prospective findings.

## Materials and Methods

The clinicohistological data from 776 patients with oral cavity lesions that included 647 lesions of a 10-year retrospective study (1993 – 2003) and 129 lesions of a one-year (2003 – 2004) prospective study, was studied. In the retrospective group the data was collected from the archives of the Departments of Pathology, Surgery, and ENT. Clinical details, personal history, and habits were collected manually from the records of the three departments, to complete the datasheets of the patients. Slides and blocks were taken from the archives of the Department of Pathology. All the slides were re-evaluated and results noted according to the definitions used in the assessment of the prospective cases.

In the prospective group, a detailed clinical work up was done for each patient. To confirm the clinical diagnosis, a biopsy was taken from the representative area. All the biopsy sections were stained with Hematoxylin and Eosin. van Gieson's stain was used to highlight the fibrous tissue in cases of submucous fibrosis.

### Statistics

Mean and standard deviation were calculated and student's ‘t’ test and kappa coefficient of agreement were used as and when required to find the significance. Values ≤ 0.05 were taken as the critical level of significance.

## Results

Various lesions included in the present study, their respective percentage, and mean age of presentation are given in Table 1. The commonest lesion in both the retrospective (RS) and prospective studies (PS) was squamous cell carcinoma.

**Table 1:** Various lesions of oral cavity (Retrospective and prospective)

Lesions	Retrospective study (10 years)			Prospective study (1 year)		
		
	No. of cases	%	Mean age at presentation	No. of cases	%	Mean age at presentation
Premalignant lesion						
Leukoplakia	67	10.36	35.79 + 15.74	11	8.53	33.68 + 11.92
Submucous fibrosis	43	6.65	35.94 + 11.23	25	19.38	33.9 + 16.41
Papillomatous lesions	64	9.89	39.04 + 30.12	12	9.30	33.29 + 22.21
Neoplasms						
Squamous cell carcinoma	389	60.12	53.15 + 12.93	53	41.09	53.42 + 11.38
Neoplasms other than squamous cell carcinoma	4	0.62	49.05 ± 8.62	4	3.10	50.31 ± 7.31
Miscellaneous						
Congenital malformations	12	1.85	5.67 ± 3.28	4	3.10	6.24 ± 3.75
Pseudoneoplastic inflammatory lesions	68	10.51	28.24 ± 10.24	20	15.50	24.56 ± 12.35
Total	647	100		129	100	

Table 2 shows the year-wise incidence rate per thousand population, surveyed in the retrospective and prospective studies. The incidence of oral cavity lesions increased to 4.31 in the years 2003 – 2004 as compared to 2.45 in the years 1993 – 1994. The increase in the incidence rate was observed in all the premalignant and malignant lesions, but the rise was most marked for oral submucous fibrosis (0.05 vs. 0.83).

**Table 2:** Year-wise incidence rate of oral cavity lesions per thousand population

Year	Population at risk	Leukoplakia		OSMF		Papillomatous lesions		Squamous cell carcinoma		Neoplasms other than squamous cell carcinoma		Miscellaneous		Total	Total %
1993-1994	17592	4	0.23	1	0.05	3	0.17	28	1.59	-	-	7	0.40	43	2.45
1994-1995	18713	5	0.27	2	0.11	3	0.16	29	1.55	-	-	5	0.27	44	2.36
1995-1996	21942	6	0.27	2	0.09	5	0.23	32	1.46	-	-	6	0.27	51	2.32
1996-1997	20853	5	0.24	3	0.14	4	0.19	36	1.73	1	0.05	7	0.34	56	2.69
1997-1998	23621	7	0.30	4	0.17	7	0.30	43	1.82	-	-	8	0.34	69	2.93
1998-1999	22224	5	0.22	3	0.13	6	0.27	40	1.80	1	0.04	8	0.36	63	2.82
1999-2000	24698	8	0.32	5	0.20	9	0.36	45	1.82	-	-	6	0.24	73	2.94
2000-2001	23508	8	0.34	6	0.26	8	0.34	44	1.87	-	-	9	0.38	75	3.19
2001-2002	25104	9	0.36	8	0.32	8	0.32	45	1.79	2	0.08	11	0.44	83	3.31
2002-2003	26802	10	0.37	9	0.34	11	0.41	47	1.75	-	-	13	0.49	90	3.36
2003-2004	30002	11	0.37	25	0.83	12	0.4	53	1.77	4	0.13	24	0.8	129	4.31

The most common clinical presentation of patients with oral cavity lesions in both RS and PS was ulceration. Growth (cases of squamous cell papilloma and carcinoma), white patch (in leukoplakia), trismus, inability to open mouth, and protruding tongue (Oral Submucous Fibrosis), dental problems, and stomatitis were some of the other presentations of oral cavity lesions.

### Leukoplakia

Seventy-eight cases of leukoplakia were studied. Patients with leukoplakia presented at an earlier age in the prospective study (33.68 ± 11.92) than in retrospective study (35.79 ±15.74), but the difference was not statistically significant. The commonest site of presentation was the buccal mucosa in both PS and RS (55% and 54%).

### Submucous firbosis

Sixty-eight cases of submucous fibrosis were studied. The commonest age of occurrence in the RS was 31 – 40 years, while in the PS it was 21 – 30 years. However, no significant difference could be found in the mean (SD) age of the RS and PS groups (35.94 ± 11.23 vs, 33.9 ± 16.41). An increase in incidence from 6.65% in RS to 19.38% in PS was also seen. The yearly incidence also showed a significant increase from 0.05 in the year 1993 – 1994 to 0.83 in the year 2003 – 2004 (*P* < 0.001). The commonest site of occurrence was the buccal mucosa in both PS and RS (56% for both).

### Squamous cell papilloma

The mean age of patients with squamous cell papilloma in RS was higher than in PS (*P* < 0.05) [Table 1]. The commonest site of presentation was the buccal mucosa, in both PS and RS (55%).

### Squamous cell carcinoma

The maximum number of cases was seen in the sixth decade (50 – 59 years) in both PS and RS [Table 1]. The commonest site of presentation was the tongue in RS and the buccal mucosa in PS. Prevalence of squamous cell carcinoma in RS was 60.12%. This decreased to 41.09% in PS. The yearly incidence showed a constant increase from 1993 – 1994 (1.59) to 2000 – 2001 (1.87), which decreased to 1.77 in the year 2003 – 2004.

### Neoplasm other than squamous cell carcinoma

Few neoplasms other than squamous cell carcinoma of the oral cavity were observed in our study. These included primary lymphoma of the tonsil, papillary adenocarcinoma, acinic cell carcinoma, and adenoid cystic carcinoma of the minor salivary glands, hemangioendothelioma (tonsil), ameloblastoma, and melanoameloblastoma were seen.

### Miscellaneous lesions

One hundred and four miscellaneous lesions, which included congenital malformations, hemangiomas, and lymphangiomas (16 cases), inflammatory pseudoneoplastic lesions (74), inflamed ectopic salivary glands (13), and malakoplakia cheek (1), were observed.

Table 3 shows the personal habits of patients having oral cavity lesion. An increase in intake of all oral intoxicants, alcohol, smoking, and even hukka was observed in PS as compared to RS. Maximum cases (34.16%) in RS had the habit of tobacco chewing followed by pan masala and areca products, while in PS, pan masala consumption was more common (38.27%), followed by tobacco and areca nut consumption. A significant increase (*P* < 0.05) was also observed in consumption of alcohol in PS as compared to RS. Increased consumption was seen in both male and female patients.

**Table 3:** Personal habits of patients with oral cavity lesions

Personal habits	Retrospective study			Prospective study		
		
	Total number (%)	Males number (%)	Females number (%)	Total number (%)	Males number (%)	Females number (%)
Pan masala	152 (23.49)	107 (24.88)	45 (20.74)	37 (28.68)	31 (38.27)	6 (12.5)
Areca catechu product	144 (22.26)	92 (21.40)	52 (23.96)	28 (21.71)	15 (18.52)	13 (27.08)
Tobacco chewing	221 (34.16)	143 (35.58)	68 (31.34)	31 (24.03)	17 (29.99)	14 (29.17)
Pan	49 (7.57)	22 (5.12)	27 (12.44)	10 (7.75)	2 (2.47)	8 (16.67)
Alcohol	27 (4.17)	25 (5.81)	2 (0.92)	12 (9.30)	10 (12.35)	2 (4.17)
Smoking	11 (1.70)	10 (2.33)	1 (0.46)	5 (3.88)	3 (3.70)	2 (4.17)
Hukka	9 (1.39)	6 (1.40)	3 (1.38)	3 (2.33)	2 (2.47)	1 (2.08)
Cigar	3 (0.46)	3 (0.70)	-	2 (1.55)	1 (1.23)	-
No addiction	10 (1.55)	-	10 (4.61)	1 (0.78)	-	1 (2.08)
Not known	31 (4.79)	12 (2.79)	19 (8.76)	-	-	-
Total	647 (100)	430 (100)	217 (100)	129 (100)	8 (100)	48 (100)

On correlation of the clinical diagnosis with the histological diagnosis, out of 776 cases in the combined study (RS + PS), 740 were histologically proved (95.35%). The kappa coefficient of agreement was found to be 0.9256 (*P* < 0.001). The sensitivity, specificity, and overall diagnostic accuracy of clinical examination in diagnosing various lesions of the oral cavity, when histological diagnosis was taken as gold standard are as shown in Table 4.

**Table 4:** Sensitivity, specificity and over all diagnostic accuracy of clinical evaluation when histology was taken as gold standard

Lesion	Sensitivity	Specificity	Overall diagnostic accuracy
Leukoplakia	82.0	99.4	97.7
OSMF	97.0	99.7	99.5
Sq. papilloma	88.2	99.4	98.5
Sq. cell carcinoma	99.5	97.9	98.8
Other inflammatory conditions	95.5	98.4	98.1

## Discussion

In the present clinicopathological study of various oral cavity lesions an effort was made to diagnose the lesion with usefulness of clinical evaluation. The present study shows the regular increase in the incidence of oral cavity lesions in the past 11 years. From 2.45 in the year 1993 – 1994, it increased to 4.31 in the years 2003 – 2004. This can be attributed to an increase in the use of tobacco, pan masala, Gutkha, smoking, and alcohol in our subcontinent.

Most of the patients with premalignant lesions (leukoplakia, oral submucous fibrosis, squamous cell papilloma) were seen in the fourth decade, whereas, Scott *et al*.(5) and Waldern and Shafer(6) in their study found the sixth and seventh decades as the commonest age of occurrence.(5,6) Besides, in a premalignant lesion, the mean age of the patients in PS was less than that in the RS, which was probably due to a decrease in the age of patients taking gutkha/pan masala, smoking, early screening, and increased awareness about the problem. Despite a decrease in tobacco consumption, the mean age of presentation for squamous cell carcinoma was 53.15 + 12.93 in RS and 53.42 ± 11.38 in PS; which was similar to previous reports from Allahabad and other parts of the country,(7–9) and may be attributed to the increased intake of pan masala and other mouth fresheners containing beetle nut (Aracacia) in place of tobacco. The number of pans chewed is also quite high (10 – 15/day) in this region. It may act as a chronic irritant to the buccal mucosa and tongue.

Leukoplakia, squamous papilloma, and squamous cell carcinoma were more prevalent in males, while oral submucous fibrosis predominantly affected females. These findings were consistent with the previous reports(8–11) and may be attributed to the increased intake of pan masala and other mouth fresheners containing beetle nut (Aracacia) by females as compared to tobacco by males.

The commonest site of presentation of premalignant lesions was the buccal mucosa in both RS and PS. Buccal mucosa was also the commonest site of presentation for squamous cell carcinoma in PS, but in RS, the tongue was the commonest site. This was in accordance with the studies of various other workers(6,11–14) The difference in the site may be attributed to the difference in causative factor (tobacco chewing or pan masala). A decrease in the incidence of squamous cell carcinoma observed in PS as compared to RS may be attributed to the increased detection of patients in premalignant stages, because of early and correct screening.

The maximum number of patients with oral cavity lesions in RS had the habit of tobacco chewing, while in PS, the habit of pan masala dominated tobacco chewing. Our findings compared well with the observations of some of the other workers.(15–18) Ramanathan(19) suggested the mechanism of tobacco and alcohol in carcinogenesis. In the present study also, alcohol consumption was found to be increased in PS and was considered to be a contributing etiological factor. Sundstrom *et al*.(20) found an association of verrucous carcinoma with tobacco chewing.

Of the total of 776 cases, 740 had clinicohistological correlation; and the percentage agreement was 95.36. The kappa coefficient of agreement was 0.9256, which was highly significant.

The main limitations of the study were that only patients reporting to the hospital or nearby clinics were included. Although it reflected a change in the pattern of various diseases, a population-based field study may give better results. Lack of cooperation from the patients in giving a history of personal habits and addiction (tobacco, smoking, pan masala, etc.) was the main confounding factor that was taken care of by a detailed history and patient counseling.

It is concluded that the incidence of premalignant lesions of the oral cavity is increasing and showing a predilection for younger age groups, due to the increase in intake of pan masala and other related intoxicants. It is like a smoldering volcano, which if not taken care of, may erupt, leading to increased morbidity and mortality. A detailed clinical work up with histology can help in diagnosing more than 95% of the cases of oral cavity premalignant lesions and reducing morbidity and mortality due to malignant lesions.
